# The Presence of EGFR T790M in TKI-Naïve Lung Cancer Samples of Patients Who Developed a T790M-Positive Relapse on First or Second Generation TKI Is Rare

**DOI:** 10.3390/cancers14143511

**Published:** 2022-07-19

**Authors:** Weiting Li, Klaas Kok, Geok Wee Tan, Pei Meng, Mirjam Mastik, Naomi Rifaela, Frank Scherpen, T. Jeroen N. Hiltermann, Harry J. M. Groen, Anthonie J. van der Wekken, Anke van den Berg

**Affiliations:** 1 Department of Pathology and Medical Biology, University of Groningen, University Medical Centre Groningen, 9713 GZ Groningen, The Netherlands; w.li@umcg.nl (W.L.); g.w.tan@umcg.nl (G.W.T.); pei.meng@med.lu.se (P.M.); m.f.mastik@umcg.nl (M.M.); n.b.rifaela@umcg.nl (N.R.); f.j.g.scherpen@umcg.nl (F.S.); 2 Department of Genetics, University Medical Center Groningen, 9713 AV Groningen, The Netherlands; 3 Department of Pulmonary Diseases, University of Groningen, University Medical Center Groningen, 9712 CP Groningen, The Netherlands; t.j.n.hiltermann@umcg.nl (T.J.N.H.); h.j.m.groen@umcg.nl (H.J.M.G.); a.j.van.der.wekken@umcg.nl (A.J.v.d.W.)

**Keywords:** NSCLC, EGFR, TKI, T790M, ddPCR

## Abstract

**Simple Summary:**

Treatment outcomes for non-small cell lung cancer (NSCLC) patients have significantly improved since the introduction of targeted therapy using tyrosine kinase inhibitors (TKI). Despite the initial promising results, most patients develop resistance due to on- or off-target mechanisms. On-target mutations prevent the effective binding of TKIs. In this study, we determined whether the most common on-target resistance mutation for treatment with EGFR-TKI mutation, i.e., the EGFR T790M mutation, can be detected in pre-treatment tissue samples and can predict treatment outcome. We included 33 patients who progressed with a T790M positive relapse upon treatment with EGFR-TKI between 2013 to 2019. Progression-free survival (PFS) time of the single T790M positive patient was 9 months, while the T790M negative patients had a median PFS of 10 months (range 2–27). Our results show that the presence of EGFR T790M mutations in pre-TKI samples is rare, even in patients who subsequently progressed with an EGFR T790M mutation.

**Abstract:**

EGFR-mutated non-small cell lung cancer (NSCLC) patients can be effectively treated with tyrosine kinase inhibitors (TKI) but frequently present with an EGFR T790M resistance mutation at relapse. We aimed to screen for T790M in pre-treatment formalin-fixed and paraffin-embedded (FFPE) tissue samples of patients with a confirmed T790M mutation at progression. We analyzed 33 pre-treatment DNA samples of NSCLC patients who progressed upon TKI between 2013 to 2019. To establish storage-time dependent formalin fixation-induced background levels for C>T mutations, we analyzed DNA isolated from archival (stored >1 year, *n* = 22) and recently generated (stored <1 month, *n* = 11) FFPE samples and included DNA isolated from white blood cells (WBC) (*n* = 24) as controls. DNA samples were analyzed by droplet digital (dd)PCR, and positivity was defined by outlier detection according to Grubb’s criterion. The T790M background allele frequency levels were 0.160% in DNA isolated from archival-FFPE, 0.100% in fresh FFPE, and 0.035% in WBC. Progression-free survival (PFS) time of the single T790M positive patient was 9 months, while T790M negative patients had a median PFS of 10 months (range 2–27). Proper storage time matched FFPE control samples are essential for reliable detection of T790M mutation at low VAF. The presence of EGFR T790M mutations in pre-TKI samples is rare, even in patients who progressed with EGFR T790M mutations.

## 1. Introduction

Lung cancer is a heterogeneous malignancy with more than 50 subtypes [1]. Non-small cell lung cancer (NSCLC) accounts for approximately 85% of all cases, and patients treated with chemotherapy or surgery have a 5-year survival rate of less than 15% [2]. Lung adenocarcinoma (LUAD) is a histomorphological subtype of NSCLC comprising about 70% of all lung cancers [3]. A subgroup of about 10% of LUADs is characterized by activating mutations in tyrosine kinases, and these patients can be effectively treated with specific tyrosine kinase inhibitors (TKI) in the Netherlands [4,5]. Patients with activating mutations in the epidermal growth factor receptor (EGFR), such as the commonly observed exon 19 deletions (E19del) and the L858R mutation, achieve a progression-free survival (PFS) time of 10–14 months upon treatment with EGFR-TKIs [6,7,8,9]. First-generation EGFR inhibitors such as gefitinib [10] and erlotinib [11] and second-generation EGFR inhibitor afatinib [12] are regarded to be successful treatments, but most if not all patients develop resistance due to on-target or off-target mutations [13]. The EGFR T790M mutation detected in about 50–60% of the recurrent tumors in patients treated with a first- or second-generation EGFR TKI is the most commonly observed on-target resistance mechanism [14,15]. With the development of third-generation TKIs, many of the on-target resistance mechanisms, including the well-known T790M, can be subsequently treated with success.

It has been suggested that in a subset of cases, the resistant mutation is already present as a minor clone in treatment-naïve tumors, while in others outgrowth of a resistant subclone is induced by the selective pressure of the TKI treatment [14,15]. Several studies have addressed the question of whether the presence of the T790M resistance mutation in treatment-naïve LUAD carrying an activating EGFR mutation may be associated with a shorter PFS. Overall, T790M has been detected at low variant allele frequencies (VAF) ranging from 0.1% to 0.5% [16,17,18,19,20,21]. In several studies, the presence of T790M in pre-treatment samples has been associated with shorter survival [17,22]. However, some studies show conflicting data with either a longer PFS [16] or no correlation with PFS [23] for T790M positive cases. A drawback in many of these studies is that the observed resistance mechanism at progression has not been determined, making it hard to establish a true causal link. Moreover, a potential pitfall for detection of the c.2369C>T p.(T790M) mutation in formalin-fixed and paraffin-embedded (FFPE) tissue samples is the high background of C>T mutations induced by formalin fixation [24,25].

Here we report the T790M analysis in EGFR TKI-naïve FFPE samples using the highly sensitive ddPCR method in patients who developed a T790M dependent resistance after treatment with first- or second-generation EGFR TKIs. To discriminate between true positive and background signals in the ddPCR, we analyzed control DNA samples isolated from blood cells, recently prepared (<1 month) and archival (>1 year) FFPE blocks for C>T and non-C>T variants. The presence of T790M in pre-treatment patient samples was established based on storage time-matched control FFPE blocks. Moreover, we aimed to correlate the PFS of baseline T790M positive and negative patients to establish a potential effect of this resistance mutation on survival.

## 2. Materials and Methods

### 2.1. Patient Selection and Control Samples

**Figure 1:**
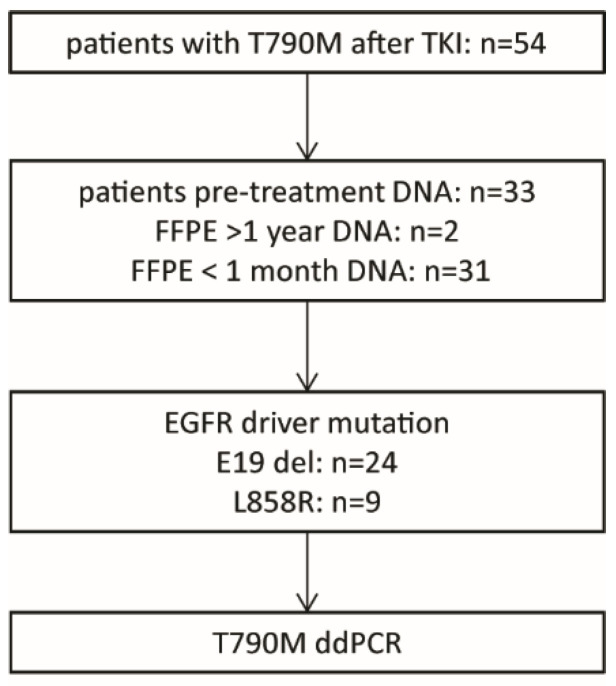
Flowchart of the patient selection strategy applied in this study.

A total of 54 patients treated with an EGFR-specific TKI and presenting with a recurrent tumor carrying the T790M resistance mutation were identified from 2013 to 2019 in the medical records of the University Medical Center Groningen (Figure 1). Of these, DNA isolated from pre-treatment FFPE biopsies for routine diagnostic testing was available in the molecular diagnostics (MD) archive for 33 patients. Control DNA samples were obtained from the MD archive of archival FFPE blocks stored >1 year before DNA isolation (*n* = 22), from FFPE blocks stored <1 month (*n* = 11) and from non-patient white blood cells (WBC) (*n* = 24) (Supplementary Figure S1). Genomic DNA of FFPE samples was extracted from the FFPE specimens using the QIAamp DNA FFPE tissue kit (Qiagen, Hilden, Germany), according to the manufacturer’s instructions. For each sample, a tumor-rich area was macro-dissected to ensure a sufficiently high tumor cell content. The minimal estimated tumor cell percentage was set at 20%. DNA from WBC was isolated using the ReliaPrep Large Volume HT gDNA Isolation kit (Promega, Madison, WI, USA) in combination with the Hamilton StarPlus robotic system (Hamilton, Reno, NV, USA). The study was performed under the OncoLifes biobanking protocol (Impact of gene amplifications on survival NWMO protocol OLS027 dd 20 May 2020), for which patients gave informed consent (https://umcgresearch.org/w/oncolifes accessed on 30 May 2022).

### 2.2. Droplet Digital (dd)PCR

We applied ddPCR to measure the number of wild-type and mutant EGFR allele copies in control and patient DNA samples according to the protocol provided by the manufacturer (Biorad, Hercules, CA, USA). Sequences of primers and probes used for the detection of the EGFR c.2369C>T (p.T790M), c.2573T>G (p.L858R), E19del (drop-off assay allowing the detection of all E19del variants), KRAS c.35G>T (p.G12V) and c.35G>A (p.G12D) mutations and their wild type counterparts are listed in Supplementary Table S1 and were ordered from Integrated DNA Technologies (IDT, Leuven, Belgium). The reaction mixes were placed in a QX200 droplet generator (Biorad, Hercules, CA, USA) and transferred to 96-well plates. Amplification reactions were done using an initial denaturation step at 95 °C for 10 min, which was followed by 39 cycles with incubation steps at 94 °C for 30 s, at the indicated annealing temperatures (Table S1) for 1 min, and a final extension step at 72 °C for 10 min, which was followed by cooling down to 4 °C. After amplification, fluorescence signals of individual droplets were detected with a QX200 droplet reader and analyzed using the QuantaSoft Analysis Pro software (Biorad, Hercules, CA, USA). In each run, multiple positive and negative control samples were included and used to set appropriate cut-off values for wild-type and mutant probes.

The number of wild-type and mutant allele copies were calculated per well, considering the Poisson distribution to correct for double positive droplets as recommended by the manufacturer. VAFs were calculated per well to establish the variation in VAF relative to the total number of allele copies analyzed. Next, VAFs were calculated per sample to determine the background for different DNA sources and different ddPCR assays. For samples with sufficient DNA, we performed multiple independent experiments with three independent wells per experiment. VAFs of samples were calculated based on the combined total number of mutant allele copies from all wells divided by the total number of allele copies over all wells. The DNA of patient tissue samples was analyzed in 1 to 5 independent experiments for T790M, dependent on the amount of available DNA. The ddPCRs for L858R and E19 del were performed once to establish the presence of sufficient tumor cell-derived DNA in the patient samples. DNA of control tissue samples were analyzed by ddPCR for T790M, L858R, KRAS G12V, and G12D in at least two independent experiments.

### 2.3. Statistical Analysis

For comparison of VAFs in DNA isolated from recently prepared and archival FFPE tissue blocks and blood, we used the Kruskal–Wallis test followed by a Dunn’s multiple comparisons test. We used Grubb’s criterion to determine whether the T790M VAF observed for patients was significantly higher as compared to storage time-matched FFPE control samples. Statistical analyses were performed using GraphPad Prism v 8.0. and IBM SPSS Statistics 23. *p*-values <0.05 were considered significant.

## 3. Results

### 3.1. T790M Background Levels in FFPE Tissue Samples

**Figure 2:**
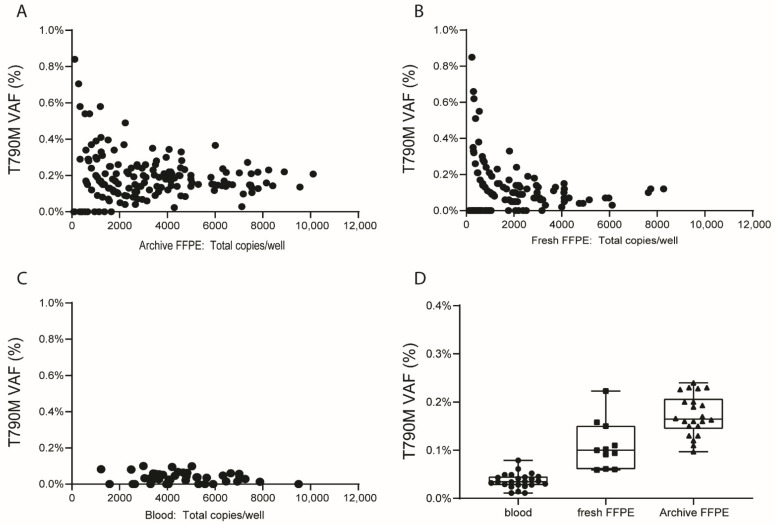
Background variant allele frequency (VAF) for the T790M mutation as assessed by droplet digital (dd)PCR. (**A**) Background VAF per well in DNA samples isolated from FFPE blocks stored >1 year. (**B**) Background VAF per well in DNA samples isolated from FFPE blocks stored <1 month. (**C**) Background VAF per well in DNA samples isolated from white blood cells (WBC). (**D**) Background T790M VAF per sample as assessed based on the sum of all mutant and wild-type probe positive droplets over all wells. Kruskall–Wallis with Dunn’s multiple comparisons test was used to establish the significance of differences observed between the three DNA sources. A *p*-value < 0.05 was considered significantly different.

To determine the variation in background levels of the c.2369C>T (p.T790M) mutation relative to the number of allele copies analyzed, we first determined VAFs at the single well level in WBCs and in tissues embedded in FFPE blocks that were stored for a short (<1 month) or a long (>1 year) period. We observed a marked variation in background VAFs which was most pronounced in DNA isolated from archive FFPE stored >1 year (Figure 2A). This was followed by a somewhat lower level of variation in DNA isolated from recently prepared FFPE blocks (stored <1 month) (Figure 2B). Overall, the variation was most pronounced for wells containing a limited number of alleles, i.e., less than 2000 for DNA isolated from recently prepared FFPE blocks and less than 4000 for DNA isolated from FFPE blocks stored >1 year. In DNA isolated from WBC, we observed overall more alleles per well, probably due to the higher quality of the DNA samples, with less variation in background T790M VAFs levels (Figure 2C). These data indicate that it is critical to analyze sufficient allele copies to obtain reliable VAFs. As a next step, we calculated the VAF for each sample by using the sum of wild-type and mutant allele copies over all wells (Table 1 and Figure 2D). VAFs were significantly different between the three sources of DNA. The background VAF was lowest in DNA isolated from WBC with a median of 0.035% (IQR: 0.028–0.044%), followed by DNA isolated from recently prepared FFPE blocks with a median of 0.100% (IQR: 0.076–0.130%), and highest in DNA isolated from FFPE blocks stored >1 year with a median of 0.160% (IQR: 0.152–0.202%).

As an additional step in our analyses, we analyzed whether the relatively high background VAFs were specific to the T790M C>T mutation or not. For the KRAS G12D C>T mutation, the VAFs observed in the three sources of DNA were similar to those observed for T790M, with a median of 0.034% (IQR: 0.015–0.057%) for WBC, a median of 0.070% (IQR: 0–0.140%) for FFPE stored <1 month and a median of 0.160% (IQR: 0.110–0.231%) for FFPE blocks stored >1 year (Table 1). For both non-C>T ddPCR assays, VAF was close to 0% in all control samples, irrespective of the sample source (Table 1 and Supplementary Figure S1). These data show that background VAF is consistently higher for C>T mutations as compared to non-C>T mutations.

Based on these combined experiments, we decided to use storage-time matched FFPE blocks to determine whether T790M VAFs were significantly higher than the background VAF, and we aimed to analyze a total of at least 2000 alleles for each patient to achieve a reliable T790M VAF measurement.

### 3.2. Patient Characteristics and Detection of T790M in Pre-Treatment Samples

Characteristics of the 33 patients presenting with a T790M at progression and with available DNA from baseline tumor samples are shown in Table 2. The median age of the patients at diagnosis was 61 years (range 38–81) and included females (*n* = 17) and males (*n* = 16). Twenty-four patients carried an E19del and nine patients an L858R EGFR driver mutation. Patients were treated with gefitinib (*n* = 17), erlotinib (*n* = 10) or afatinib (*n* = 6). Median PFS was 10 months (range 2–27) for first-line EGFR-TKI therapy. The presence of a T790M mutation at progression was shown by routine molecular testing of the relapse sample using targeted NGS.

**Figure 3:**
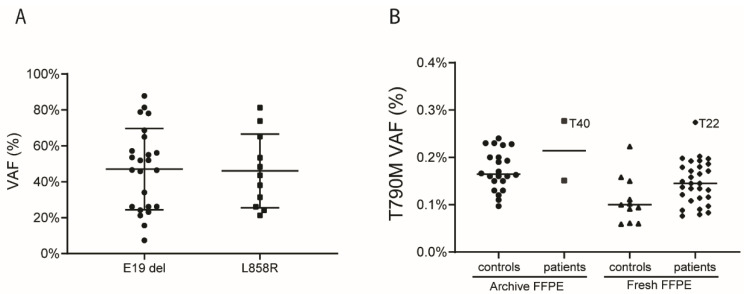
Overview of EGFR E19del, L858R, and T790M variant allele frequencies (VAFs) as assessed by droplet digital (dd)PCR. (**A**). VAF of the EGFR driver mutations in the 33 selected patients. (**B**) T790M VAF in controls and patients split based on storage time of the FFPE blocks. Archive FFPE, storage time of FFPE blocks before isolation of DNA >1 year; fresh FFPE, storage time of FFPE blocks before isolation of DNA <1 month. Grubb’s criterion was applied to determine whether individual patients were outliers relative to the storage time-matched controls. Grubb’s criterion resulted in a VAF cut-off value of 0.228 and 0.289 for DNA isolated from FFPE blocks stored for a short and long time, respectively. Patient T22 was positive, while patient T40 was just below the cut-off.

DNA from these 33 EGFR-TKI naïve NSCLC patients was first evaluated for tumor content by determining the VAF of the EGFR driver mutation (E19 del and L858R) by ddPCR. The median VAF was 49.2% for E19del (range 7.3% to 87.8%) and 43.6% for L858R (range 21.3% to 81.2%), indicating the presence of tumor cell DNA in all samples (Figure 3A, Table 2 and Table S2). For 31 out of 33 patients, DNA was isolated from FFPE blocks stored for <1 month, while for 2 patients, DNA was isolated from FFPE blocks stored for >1 year. Analysis of multiple wells and multiple experiments per patient revealed a median number of total allele copies analyzed per patient of 17,311 (IQR: 9499 to 31,690). The median T790M VAF in the patients with DNA isolated from FFPE blocks stored for <1 month was 0.145% (IQR: 0.114–0.180%). For the two patients with DNA being isolated from FFPE blocks with a long storage time, the VAF for T790M was 0.151% and 0.277%. Based on Grubb’s criterion, the T790M VAF of patient T22 was an outlier (1 out of 33 patients, 3% of the patients) (Figure 3B and Table S2). For a second patient (T40), the T790M VAF was very close but just below the upper limit according to Grubb’s criterion. For all other patients, the T790M VAF was not significantly different from those observed in storage-time matched FFPE controls. The observed T790M VAFs did not correlate with the VAF of the activating mutation (Table S2).

The PFS was 9 months for patient T22, and 10 months for patient T40 with a VAF close to the cut-off level. The PFS of the 31 T790M-negative patients ranged from 2 to 27, with a median PFS of 10 months. Thus, no differences were found between the PFS of patients with and without T790M in the pre-treatment samples in our patient group.

## 4. Discussion

In this study, we focused on the assessment of T790M positivity in EGFR-TKI pre-treatment samples of patients who developed a T790M mutation at disease progression. Our data show that before treatment, the frequency of T790M positivity is very low (1/33) even in this selected group of patients who all developed a T790M positive relapse at progression. Therefore, the mechanism for resistance in patients relapsing with a T790M positive tumor under TKI pressure depends on the outgrowth of new subclones and not on the outgrowth of pre-existing T790M positive subclones.

The ddPCR technique is regarded to be a very sensitive approach, allowing detection of VAF as low as 0.1% or even lower (0.01% [15] or 0.001% [26]). However, the actual sensitivity of the ddPCR is dependent on the number of allele copies studied per sample. Single or low numbers of positive droplets can potentially result in a relatively high VAF when low numbers of total allele copies are analyzed. Due to the frequency of small lung cancer biopsies available for diagnostic testing, the available amount of DNA is limited, and this precludes a reliable assessment, especially of variants present at low VAFs. A second factor influencing background VAFs in DNA isolated from FFPE tissue blocks is caused by formalin fixation, which leads to the deamination of cysteine nucleotides and subsequent incorporation of adenosine residues instead of guanine residues during the PCR [25]. We showed a clear difference between the background VAFs for two C>T variants compared to two non-C>T variants in DNA isolated from FFPE blocks, which was most pronounced in FFPE blocks stored for >1 year prior to DNA isolation. By determining VAFs per well, we showed that the variation in background VAFs is high, especially in wells with a limited number of analyzed allele copies and in DNA isolated from FFPE blocks stored for >1 year. Analysis of more than 2000 copies was enough to reduce the variation in VAFs for FFPE blocks stored for a short time prior to DNA isolation, while a total of more than 4000 copies was required for FFPE blocks stored for >1 year. These results indicate that analyzing sufficient allele copies is critical, especially when expecting low VAFs (<0.5%), and this can be achieved by pooling measurements of multiple wells and multiple independent experiments. Based on Grubb’s criterion, we used VAF cut-off values of 0.228 and 0.289 for DNA isolated from FFPE blocks stored for a short and long time, respectively. In the literature, VAF cut-offs used to discriminate between positive and negative cases varied from 0.1% to 0.5%, with a minimum of at least 2, 6, or 10 positive droplets per well [16,17,18,19,20,21,22]. Based on our control samples, 0.1% appears not to be discriminative, while 0.5% might be too strict. However, we cannot exclude variation in reliable cut-off values between different laboratories. Moreover, for most studies, the number of allele copies studied per sample is unclear.

Several studies have analyzed the presence of T790M in EGFR TKI naïve patients. In earlier studies, up to 13% of pre-TKI samples of EGFR driver mutation-positive patients were shown to be positive for T790M using techniques other than ddPCR [27]. In one of the first large ddPCR studies, close to 80% of the patients were found to be positive, but most cases had a VAF < 0.1% [25]. The authors indeed proposed that most of these cases should be considered negative [27,28]. Two smaller studies reported a relatively high frequency of T790M in pre-treatment samples, but also in these studies, the authors indicated that the percentage of positive samples would be much lower with more strict criteria [18,21]. In two recent larger studies, the frequency of T790M positive samples was less than 5% [20,21]. We also observed a low frequency of T790M positive patients, with 1 out of 33 being positive, like the two large studies. A strong point of our study is that we focused on patients who presented with T790M at progression, so we excluded patients with a resistance mechanism other than T790M. Despite this selection, the incidence of T790M positive pre-treatment samples was low.

We cannot rule out that the failure to detect T790M in treatment-naive patients is related to the heterogeneity of the tumor. The biopsy that was analyzed might not have contained the pre-existing T790M positive subclone that was responsible for the relapse. A potentially more effective strategy to detect pre-existing T790M positive subclones might be by ddPCR analysis of circulating cell-free (cf)DNA. This has the potential to circumvent the problem of intratumor heterogeneity. Good consistency between the presence of T790M in tissue and cfDNA has been shown in previous studies [19,29,30]. In a study focusing on the detection of T790M in longitudinal cfDNA samples, the authors showed good consistency between clinical response and T790M VAF [18]. However, a negative cfDNA test does not rule out the presence of T790M in the tumor [31]. Overall, this indicates that parallel screening of tissue and cfDNA can potentially increase sensitivity to detect all T790M positive patients at progression.

The presence of T790M in exon 20 of the EGFR gene reduces the binding of first- and second-generation EGFR TKIs by enhancing the binding affinity of ATP to the kinase domain of the EGFR-mutant receptor [32]. This mutation accounts for acquired resistance in about 50% to 60% of the patients treated with first or second-generation TKIs [33,34]. The T790M-positive patient in our study had the highest VAF for the EGFR driver mutation (88%), while the borderline positive patient had a slightly lower VAF (65%). On the one hand, a high driver-VAF might indicate a high tumor cell content, which might increase the chance of detecting the putative T790M positive subclone in the pre-TKI sample. On the other hand, a high driver-VAF might also indicate the presence of EGFR copy number gain in tumor cells, which would not lead to an increased chance of detecting a putative T790M positive subclone. We previously showed that EGFR copy number gain was associated with shorter overall survival time but not with PFS [35]. This might be related to previous observations that EGFR copy number gain is a resistance mechanism for osimertinib [36]. Third-generation EGFR TKIs like osimertinib are effective in the treatment of these T790M-positive patients. In Western countries, most EGFR-mutant patients are nowadays treated with Osimertinib as a first-line EGFR TKI treatment. However, as the less expensive first- and second-generation TKIs are still in use in many (developing) countries, this study remains relevant [37,38,39,40]. Moreover, our finding that a pre-existing T790M resistant mutation is very rare may be generalized to other TKI-related resistance mutations. 

PFS of pre-treatment T790M negative patients in our study treated with first- or second-generation EGFR TKIs ranged from 2 to 27 months with a median of 10 months. Most papers showed shorter PFS in pre-treatment T790M-positive patients. Patients with negative or low T790M VAF had a PFS of 16.8 and 14 months, respectively, while patients with high T790M VAF had a PFS of 6.2 months [22]. In another study, the PFS was 13.1 months in T790M negative patients and 8.5 months in T790M positive patients [21]. Similarly, PFS of 11.5 and 6.3 months were observed in T790M negative and positive patients, respectively [41]. In contrast, PFS was reported to be longer in T790M positive patients compared to T790M negative patients, 29.2 months versus 17.7 months [28]. The single pre-TKI T790M positive patient in our study had a PFS of 9 months, while the borderline positive patient had a PFS of 10 months. This indicates that in our study presence of a T790M positive subclone in pre-TKI tumor samples was not associated with PFS. In a previous study including a limited number of patients, the authors proposed a model in which pre-treatment of T790M negative or weakly positive tumor samples might result in rapidly growing T790M-independent relapses, while pre-treatment samples with high T790M VAF would lead to more slowly growing T790M-dependent relapses [42]. In contrast to our study, previously published studies did not focus on patients with a proven T790M-dependent relapse. 

## 5. Conclusions

We report an optimized approach to analyze ddPCR data allowing a more reliable measurement of low T790M VAFs as expected in pre-treatment FFPE samples. Moreover, to the best of our knowledge, this is the first study to focus specifically on patients with T790M as the proven resistance mutation. Despite this selection, the frequency of pre-TKI-positive patients using tissue biopsies remains low, with 1 out of 33 patients being positive. So, testing for T790M in pre-TKI tissue samples seems not to be effective in predicting clinical response to first and second-generation EGFR-TKIs in a clinical setting

## Figures and Tables

**Table 1:** Results of the ddPCR tests on DNA isolated from WBC and FFPE blocks stored for a short (<1 month) and long time (>1 year).

ddPCR Assay	Sample Group	Median VAF (%)	IQR	*p*-Value
EGFR T790M C>T	archive FFPE	0.160	0.145–0.207	P_overall_ < 0.001 (archive vs. blood < 0.001; archive vs. fresh 0.003)
	fresh FFPE	0.100	0.061–0.150	
	Blood	0.035	0.028–0.045	
KRAS G12D C>T	archive FFPE	0.158	0.071–0.220	P_overall_ < 0.001 (archive vs. blood < 0.001)
	fresh FFPE	0.047	0.010–0.090	
	Blood	0.020	0.016–0.025	
KRAS G12V nonC>T	archive FFPE	0.000	0.000–0.074	NS
	fresh FFPE	0.000	0.000–0.000	
	Blood	0.000	0.000–0.008	
EGFR L858R nonC>T	archive FFPE	0.000	0.000–0.006	NS
	fresh FFPE	0.000	0.000–0.002	
	Blood	0.000	0.000–0.011	

**Table 2:** Clinical characteristics of the 33 patients with T790M at the progression of EGFR-TKI.

Patient Characteristics	Number/Frequency
Gender (male/female)	16/17
Age (median (range)	61 (38–81)
TKI (*n*=) Gefitinib Erlotinib Afatinib	17 10 6
PFS median (range) in months	10 (2–27)
Driver mutation (*n*=) E19del L858R	24 9
Median variant allele frequency (range) (%)	47 (7–88)

## Data Availability

Data supporting the reported results can be obtained from the corresponding author.

## Supplementary Materials

The following are available online at https://www.mdpi.com/article/10.3390/cancers14143511/s1, Figure S1: Overview of control samples and results of ddPCR background assessment for one C>T variant (KRAS G12D) and two non-C>T variants (EGFR L858R and KRAS G12V), Table S1: Sequence of primers and probes used for ddPCR, Table S2: Characteristics of the 33 patients including VAF of the driver and the T790M variants.
