# Supplementary material for: The Presence of EGFR T790M in TKI-Naïve Lung Cancer Samples of Patients Who Developed a T790M-Positive Relapse on First or Second Generation TKI Is Rare
Source: Cancers (Basel). 2022 Jul 19;14(14):3511. doi: 10.3390/cancers14143511 (PMC9320221; doi:10.3390/cancers14143511)
Supplement: Supplementary file 1 [file cancers-14-03511-s001.zip › cancers-1772502-SI.pdf]

The Presence of EGFR T790M in TKI-Naïve Lung Cancer Samples of Patients Who Developed a T790M-Positive Relapse on First or Second Generation TKI Is Rare

Weiting Li [1,2], Klaas Kok [2,*], Geok Wee Tan [1], Pei Meng [1], Mirjam Mastik [1], Naomi Rifaela [1], Frank Scherpen [1], T. Jeroen N. Hiltermann [3], Harry. J. M. Groen [3], Anthonie J. van der Wekken [3] and Anke van den Berg [1,*]

[1] Department of Pathology and Medical Biology, University of Groningen, University Medical Centre Groningen, 9713 GZ Groningen, The Netherlands; w.li@umcg.nl (W.L.); g.w.tan@umcg.nl (G.W.T.); pei.meng@med.lu.se (P.M.); m.f.mastik@umcg.nl (M.M.); n.b.rifaela@umcg.nl (N.R.); f.j.g.scherpen@umcg.nl (F.S.)

[2] Department of Genetics, University Medical Center Groningen, 9713 AV Groningen, The Netherlands

[3] Department of Pulmonary Diseases, University Medical Center Groningen, University of Groningen, 9712 CP Groningen, The Netherlands; t.j.n.hiltermann@umcg.nl (T.J.N.H.); h.j.m.groen@umcg.nl (H.J.M.G.); a.j.van.der.wekken@umcg.nl (A.J.v.d.W.)

* Correspondence: k.kok@umcg.nl (K.K.); a.van.den.berg01@umcg.nl (A.v.d.B.)

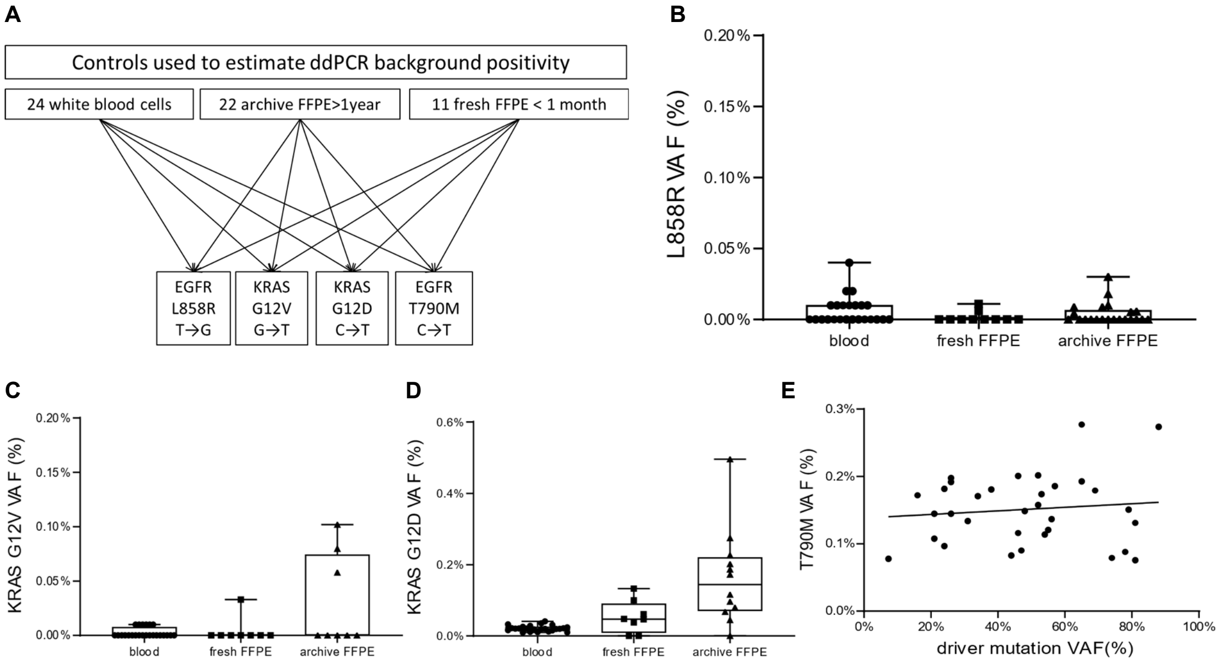

Figure S1. Overview of control samples and results of ddPCR background assessment. **A**. Overview of the control samples and ddPCR assays used to determine the background VAFs. **B.** Results of the L858R ddPCR in DNA isolated from WBC, archive and fresh FFPE blocks **C.** Results of the ddPCR for KRAS G12V. **D.** Results of the ddPCR for KRAS G12D. Archive FFPE, storage time of FFPE blocks before isolation of DNA >1 year; fresh FFPE, storage time of FFPE blocks before isolation of DNA <1 month. Kruskall Wallis with Dunn's multiple comparisons test was used to establish significance of differences observed between the three DNA sources. A p-value >0.05 was considered significantly different. WBC, white blood cells; archive FFPE, FFPE blocks stored <1 month; fresh FFPE, FFPE blocks stored >1 year. **E** The VAF of the driver mutation plotted versus the VAF of the T790M mutation in patient samples.

Table S1. Sequence of primers and probes used for ddPCR.

Gene	Mutation	Primer/Probe	sequence5'-3'	Dye	Length (nt)	Tm (℃)
EGFR	E19 del	FP	GTGAGAAAGTTAAAATTCCCGTC		23	60
		RP	CACACAGCAAAGCAGAAAC		19	
		WT probe	AAGGAATTAAGAGAAGCAACATCTCC	HEX	26	
		Mut probe	ATCGAGGATTTCCTTGTTGGCT	FAM	22	
	L858R	FP	GCAGCATGTCAAGATCACAGATT		23	60
		RP	CCTCCTTCTGCATGGTATTCTTTCT		25	
		WT probe	AGTTTGGCC**A**GCCCAA	HEX	16	
		Mut probe	AGTTTGGCC**C**GCCCAA	FAM	16	
	T790M	FP	GCCTGCTGGGCATCTG		16	55
		RP	TCTTTGTGTTCCCGGACATAGTC		23	
		WT probe	ATGAGCTGC**G**TGATGAG	HEX	17	
		Mut probe	ATGAGCTGC**A**TGATGAG	FAM	17	
KRAS	G12D/V	FP	GATTCTGAATTAGCTGTATCGTC		23	55
		RP	TGTGACATGTTCTAATATAGTCAC		24	
		WT probe	TTGGAGCTG**G**TGGCGT	HEX	16	
		Mut probe	TTGGAGCTG(**A/T**)TGGCGT	FAM	16	

[1] mutated nucleotides are indicated in bold in the WT and mutant probe sequences.

Table S2. Characteristics of the 33 NSCLC patients.

patient ID	FFPE storage time	driver nutation	VAF of driver mutation (%)	TKI	PFS (months)	T790M VAF (%)	Grubb's criterion: outlier[1]
T22	short	E19del	88	afatinib	9	0.274	yes
T10	short	E19del	81	erlotinib	10	0.131	no
T50	short	L858R	81	erlotinib	10	0.076	no
T16	short	E19del	78	gefitinib	7	0.088	no
T15	short	L858R	74	gefitinib	23	0.079	no
T28	short	E19del	69	gefitinib	13	0.179	no
T34	short	L858R	65	erlotinib	11	0.193	no
T12	short	E19del	57	afatinib	3	0.186	no
T18	short	E19del	56	afatinib	9	0.137	no
T38	short	E19del	55	erlotinib	27	0.121	no
T26	short	E19del	54	gefitinib	15	0.114	no
T8	short	L858R	53	erlotinib	21	0.174	no
T13	short	E19del	52	gefitinib	4	0.202	no
T27	short	E19del	52	afatinib	9	0.158	no
T19	short	L858R	48	erlotinib	14	0.149	no
T43	short	E19del	47	erlotinib	18	0.090	no
T45	short	E19del	46	gefitinib	7	0.116	no
T55	short	E19del	46	gefitinib	8	0.201	no
T49	short	L858R	44	gefitinib	10	0.083	no
T48	short	L858R	38	gefitinib	2	0.181	no

T5	short	E19del	34	erlotinib	8	0.171	no
T21	short	L858R	31	erlotinib	6	0.134	no
T23	short	E19del	26	afatinib	12	0.145	no
T1	short	E19del	26	gefitinib	19	0.192	no
T3	short	L858R	26	gefitinib	6	0.198	no
T44	short	E19del	24	erlotinib	11	0.097	no
T53	short	L858R	24	gefitinib	6	0.182	no
T54	short	L858R	21	gefitinib	22	0.145	no
T11	short	E19del	21	gefitinib	7	0.108	no
T7	short	E19del	16	gefitinib	21	0.172	no
T20	short	E19del	7.3	gefitinib	12	0.078	no
T30	long	E19del	79	gefitinib	4	0.151	no
T40	long	E19del	65	afatinib	10	0.277	no

[1] Cutoff values according to Grubb's criterion are 0.228 and 0.289 for DNA isolated from FFPE blocks stored for a short and long time, respectively.
